# Nurses’ knowledge of pressure ulcer and its associated factors at Hawassa University comprehensive specialized hospital Hawassa, Ethiopia, 2018

**DOI:** 10.1186/s12912-020-00446-6

**Published:** 2020-06-15

**Authors:** Ezedin Molla Muhammed, Berhanu Boru Bifftu, Yemataw Zewdu Temachu, Tarkie Abebe Walle

**Affiliations:** 1 Hawassa University Comprehensive Specialized Hospital, Hawssa, Ethiopia; 2 Department of Psychiatry Nursing, University of Gondar, Gondar, Ethiopia; 3 Department of Emergency Nursing, University of Gondar, Gondar, Ethiopia; 4 Department of Surgical Nursing, University of Gondar, Gondar, Ethiopia

**Keywords:** Knowledge, Nurses, Pressure ulcer prevention

## Abstract

**Background:**

Pressure ulcer is largely avoidable, but its prevalence rate increased more than 80% in a 13 years study. Nurses have a great position to advance best practices towards the prevention of pressure ulcers. Therefore they should be knowledgeable of the signs and symptoms of pressure ulcers, and preventive strategies to reduce its incidence, but there is limited evidence on nurses’ knowledge and its associated factors to prevent pressure ulcers in Ethiopia**.**

**Methods:**

A hospital-based cross-sectional study was conducted from March 25 – April 23/ 2018. A total of 356 nurses were selected by stratification with a simple random sampling technique. Pretested structured questionnaire with closed and open-ended questions was used to collect data. Frequency distribution and percentage were computed to describe each variable. Bivariate and multivariable logistic regression with a 95% confidence interval was also carried out to see the effect of each independent variable on the dependent variable and declared statistically significant association with *P* < 0.05.

**Result:**

The mean knowledge score of nurses was 25.22 out of 41 item questions. Fifty-two point 5 % of nurses score above the mean. Males [AOR = 0.44, 95% CI (0.26–0.73)], working a maximum of eight hours [AOR = 3.57, 95% CI (1.48–8.61), not having training [(AOR = 2.31, 95% CI (1.14–4.61)], Low salary [AOR = 3.47, 95% CI (1.03–11.67)] were significantly associated with inadequate knowledge.

**Conclusion:**

Generally a nurse’s knowledge of pressure ulcers was inadequate. Being female, working less than or equal to eight hours, not having the training and low working salary are contributors to a low level of knowledge for pressure ulcers.

## Background

Pressure ulcers are described as ‘localized injury to the skin and/or underlying tissue, usually over a bony prominence, as a result of pressure or pressure in combination with shear [1–4]. Pressure ulcers are classified in four stages by tissue layer affected ranging from skin erythema to damage to muscle and underlying bone, and which vary in size and severity of tissue damage [2].

A cross-sectional study conducted at Felegehiwot and Dessie referral hospital, in Ethiopia reported 16.8 and 14.9% overall prevalence rate of PU, respectively [5, 6]. While caregivers practice the best care every time, patients can avoid needless suffering [7]. Pressure area care is an essential component of nursing practice, with all patients potentially at risk of developing a pressure ulcer [8].

Annually more than 2.5 million patients develop pressure ulcers in the United States of America alone [9]. Management of hospital-acquired pressure ulcers costs billions of dollars each year; for a single individual with a diagnosis of pressure ulcer costs nearly 129,000$ on average [10]. A pressure ulcer is largely avoidable, but in a 13 years study, its prevalence rate increased more than 80% [11]. A comparative study conducted in Norwegian and Irish sites shows that PU prevalence was 54% in the Norwegian and 12% in the Irish site [12]. Another study done in Sweden revealed that the 21 different countries prevalence ranged from 9 to 31% [13]**.** A similar study conducted in Ethiopia indicates that off total among total admitted patients 16.8% of them had a pressure ulcer [5]**.**

Hospitals need to devote more resources to prevent and manage pressure ulcers. Professionals should also meet their responsibility to provide continuous nursing and medical education to staff about pressure ulcers [14].

Adequate application of incontinence management and measures are taken to prevent skin damage, such as preventive skincare based on principles of cleansing, enhancing the skin’s moisture barrier, and regular turning and repositioning along with protection [15]. Timely and accurate assessment of pressure ulcers depends on individual need, with education on skin and risk assessment forming a key component [16].

Nurses have a great position to advance best practices towards the prevention of PU. Therefore they are in need to be knowledgeable about the signs and symptoms of pressure ulcers, and preventive strategies to reduce its incidence [16]. But according to a study conducted across the globe nurses do not have sufficient knowledge about pressure ulcer prevention, classification, and management [17]. Studies in Nepal revealed that only 59% of nurses had adequate knowledge about pressure ulcer prevention [18]. A similar study conducted in North West Ethiopia shows nearly half 54.4% of the nurses had good knowledge of pressure ulcer prevention [19]**.**

The prevalence of pressure ulcers decreased if the patient is assessed for the risk of pressure ulcer upon his/her admission and if a regular assessment is followed by appropriate action or intervention [20]. Providing sufficient education, a positive attitude, and addressing barriers are all important aspects to improve the knowledge and use of pressure ulcer preventive measures among nursing staff [21]. Understands nurses’ knowledge about pressure ulcer prevention, classification, and management is important to improve their knowledge of pressure ulcer prevention.

### Justification of the study

PU is a major significant and complex problem in hospitals in terms of human suffering, tissue necrosis, pain, septicemia, disfigurement, loss of productivity, and financial burden. Nurses have typically expended most of their time with the patients. Therefore nurses have a pivotal position and role to prevent and manage pressure ulcers by correcting interdisciplinary teamwork. So nurses require complete knowledge to prevent and monitor all conditions associated with pressure ulcer occurrences.

To my best search, there is limited evidence on nurses’ knowledge and its associated factors towards pressure ulcer prevention in Ethiopia as general, and there has been no published data particularly in the study area. Therefore, information emanating from this study will be a valuable reason for the future in developing appropriate educational strategies and training in this area.

### Objectives

#### General objective

To assess Nurses’ knowledge towards pressure ulcer prevention, and its associated factors in Hawassa University Comprehensive Specialized Hospital, Hawassa, Ethiopia, 2018.

#### Specific objectives

- To determine knowledge of nurses’ on pressure ulcer prevention at Hawassa University Comprehensive Specialized Hospital, Hawassa, Ethiopia, 2018.
- To identify factors associated with the knowledge of nurses on pressure ulcer prevention at Hawassa University Comprehensive Specialized Hospital, Hawassa, Ethiopia, 2018.

## Methods

### Study design, area and period

The institution-based cross-sectional study design was conducted from March 25 – April 23/ 2018. The study was conducted in Hawassa University comprehensive specialized Hospital, Hawassa. Hawassa is found in southern Ethiopia, on the shores of Lake Hawassa in the great rift valley; 273 km South of Addis Ababa via Debre Zeit and 1125 km North of Nairobi. Currently, Hawassa town has one comprehensive specialized hospital, one regional hospital, and eight government health centers. Hawassa University Comprehensive Specialized Hospital is a teaching Hospital that gives services for more than five million people including Sidama zone and peoples of the neighboring zones and regions. In this teaching hospital, there are multidisciplinary professionals with different specialties are found, among them the big number is taken by nurses, nearly five hundred nurses currently serving in different units and office in the hospital.

### Source and study population

The source population was all nurses working at Hawassa university comprehensive specialized hospital. The study subjects were those nurses working at Hawassa University Comprehensive Specialized Hospital during the data collection period.

### Inclusion and exclusion criteria

All permanent staff nurses working at Hawassa University Comprehensive Specialized Hospital who were available during the study period were included in the study.

### Sample size determination and sampling procedure

#### Sample size

The sample size determined by using a single population proportion formula and considering the following assumptions: nurses knowledge on pressure ulcer prevention 36.15 [21]. = standard normal distribution value at 95% confidence level of Zα/2 = 1.96 and margin of error (d) = 5%.

$$ \mathrm{n}=\frac{{\left(\mathrm{Z}\upalpha /2\right)}^2\ \mathrm{P}\ \left(1-\mathrm{P}\right)}{{\mathrm{d}}^2} $$ [22]

The final sample size was determined considering a 10% non-response rate, the total sample size was 391 nurses.

#### Sampling procedures

**Fig. 1:**
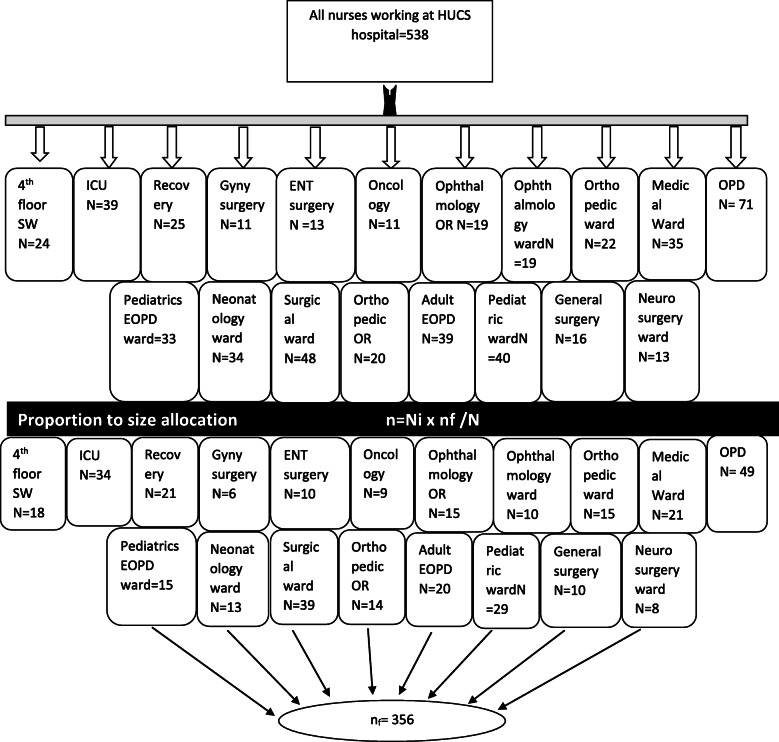
Diagrammatic representation of the sampling procedure. Where Ni = the number of nurses working at each unit/ward, nf = the final sample size (356), N = total number of nurses working at HUCSH, Ethiopia, 2018

Stratification with a Simple random sampling method was used to select the study subjects after proportional allocation for each working ward/unit. The study participants were selected from each ward/unit by a simple random sampling technique from the list of nurses in each stratum (Fig. 1).

#### Operational definitions

- **Good knowledge:** Nurses, who scored above the mean score of the knowledge questions towards pressure ulcer
- **Poor knowledge:** Nurses, who scored mean and above the mean of the knowledge questions towards pressure ulcer [19].

#### Data collection tools and procedures

Self-administer structured questionnaire containing both closed and open-ended questions were used to collect data. The data was collected from March 25 – April 23/ 2018. The question focused on Nurse’s knowledge, perceived barrier, work-related factors, and socio-demographic characteristics towards pressure ulcer prevention. Knowledge of nurses was assessed by 41 true/false questions and by using Pieper-Zulkowski Pressure Ulcer knowledge test (PZ-PUKT) [23], in its version adapted and validated to Brazil [24]. Perceived barrier questions that include un Proportionate nurse to patient ratio, Lack of guidelines, Shortage of time, Limited resource, Patient factors, Lack of evidence supported by research, Lack of training, lack of job satisfaction, Lack of knowledge were assessed by using a 5 item Likert scale (ranging from strongly agreed, 5 to strongly disagree, 1) adapted from different published literature [14, 19, 25, 26].

#### Data quality control, processing, and analysis

To keep the quality of the collected data all possible attempts were made starting from the development of a data collection instrument to completeness checking of the filled questionnaires. The questionnaire was pre-tested on 20 (5%) nurses who were working at Gondar University Comprehensive Specialized Hospital, 1 week before the initiation of the main study. And necessary corrections were made and the questions were simplified based on the pretest findings. To ensure the quality of data collection, orientation training about the data collection process was given for data collectors.

EPI-INFO version 7 statistical software was used for data entry. Then it was exported to SPSS version 20.0 for analysis. Frequency distribution and the percentage were computed to describe each variable. Binary logistic regression analysis was employed to determine the association of independent variables with the nurse’s knowledge of pressure ulcer prevention. Odds ratio with 95% confidence interval was calculated, the variables that are found with *P* < 0.2 at bivariate analysis were entered to multivariable analysis and statistical significance was declared at *p*-value less than 0.05. Finally, results were presented using tables and figures.

## Results

### Socio-demographic characteristics of respondents

Overall, among 532 nurses who were working regularly at Hawassa University Comprehensive Specialized Hospital 391 nurses were selected for the study and 356 (91.05%) responded to the question, 35 nurses refused to respond the self-administered questionnaire. Of all 185 (52%) were females. The mean age of the study subjects was 27.39 years, 80.30% were aged less than 30 years and above 39 years. The Amhara ethnic group comprised 28.7% of the study subjects followed by Oromo (14%). Nearly half of the study subjects were orthodox by religion (46.30%), followed by protestant (Table 1).

**Table 1:** Socio-demographic characteristics of nurses towards pressure ulcer prevention at Hawassa University Comprehensive Specialized Hospital, 2018

Characteristics (***n*** = 356)	Frequency	Percent
Sex	356	100.00
Male	171	48.00
Female	185	52.00
Age		
20–29	286	80.30
30–39	61	17.10
> 39	9	2.50
Ethnicity		
Sidama	46	12.90
Amhara	102	28.70
Wolaita	39	11.00
Oromo	50	14.00
Tigrie	21	5.90
Gurage	40	11.2
Others	58	16.3
Religion		
Protestant	162	45.50
Orthodox	165	46.30
Muslim	16	4.50
Catholic	5	1.40
Other	8	2.20
Marital status		
Single	190	53.40
Married	163	45.80
Divorced	2	0.60
Widowed	1	0.30
Level of Qualification		
Diploma	90	25.30
Bachelor degree	266	74.70
Total work experience		
0–4 years	168	47.20
5–10 years	163	45.80
> 10 years	25	7.00
Working ward currently		
Intensive care unit	68	19.10
Inpatient ward	149	41.90
Operation room	55	15.40
Emergency department	35	9.80
Outpatient department	49	13.80
**Variable**	**Frequency**	**Percent**
Monthly income		
< 103.2 USD	33	9.30
103.2–169.3 USD	177	49.70
169.4–251.6 USD	116	32.60
> 251.6 USD	30	8.40
Working position		
Ward head	14	3.90
Team leader	28	7.90
Focal person	24	6.70
No position/ Technical staff	290	81.50
Additional work other than this hospital		
Yes	195	54.80
No	161	45.20
Average daily duration of working time in the hospital		
≤ 8 h	169	47.50
9–12 h	127	35.70
> 12 h	60	16.90
Did you receive training about PU		
Never received training	300	84.30
Lecture	38	10.70
Course	13	3.70
Conference	2	0.60
Workshop	3	0.80
How often read literature about PU		
Never	137	38.5
Always	22	6.2
Sometimes	197	55.3

### Work-related characteristics

Among all nurses involved in the study (74.70%) have a bachelor’s degree and the rest (25.30%) were diploma holders. Forty-seven percent of the study subjects were having an experience of fewer than 5 years, (45.8%) reported they have 5–10 years and the rest (7%) were had more than 10 years of experience. Of the total nurse, 41.90% were working at the inpatient department, followed by (19.1%) intensive care unit. Experience of less than 1 year in the current ward/department accounts (53.9%), followed by 1–2 years (26.7%), and > 2 years (19.4%). Among nurses, only 33% were got less than 3201 birrs monthly salary. Towards working position 3.9% were ward head, 7.9% team leader, 6.7% focal person, and 81.5% nurses who care only the patient with no additional leadership/ward head role (Table 1).

### Nurses knowledge of pressure ulcer prevention

Among 356 nurse Participants 52.5% were scored above the mean and the rest 47.5% were scored mean and below the mean out of 41 item questions. The nurses answered correctly 57.9% (+ 1.44) of the question in the pressure ulcer classification and evaluation section. The highest rate of the correct answer was 86% (Stage IV pressure ulcers present total tissue loss, with intensive destruction and necrosis of the tissue or damage to the muscles, bones, or supporting structures) and also with 75.6% (Stage I pressure ulcers are defined as intact skin with hyperemia of a localized area and non-bleachable redness or different color from the surrounding area). The lowest rate was 16.9% (Stage II pressure ulcers present loss of dermis in its total thickness).

### Factors associated with nurse’s knowledge towards pressure ulcer

Regarding this, sex (AOR 0.56, 95% CI 0.36–0.88), working hour (AOR 2.57, 95% CI 1.17–5.61), salary (AOR 3.47, 95% CI 1.03–11.67), and training. (AOR 2.31, 95% CI 1.14–4.61) were shown to have a strong statistical association during multivariable analysis (Table 2).

**Table 2:** Factors associated with Nurses knowledge towards pressure ulcer prevention at Hawassa University Comprehensive Specialized Hospital, 2018

Variables	Nurses total knowledge score		COR (95% CI)	AOR (95% CI)
	> 25	0–25		
	N**o** (%)	N**o** (%)		
sex				
male	102 (59.60)	69 (40.40)	0.57 (0.37–0.87)^a^	0.563 (0.36–0.88)^a^
female	85 (45.90)	100 (54.10)	1.00	1.00
salary				
< 3201	13 (39.40)	20 (60.60)	6.15 (1.97–19.14)^a^	3.47 (1.03–11.67)^a^
3201–5250	84 (47.50)	93 (52.50)	4.43 (1.72–11.36)^a^	3.90 (1.42–10.68)^a^
5201–7800	66 (56.90)	50 (43.10)	3.03 (1.15–7.97)^a^	2.95 (1.04–8.30)^a^
> 7800	24 (80)	6 (20.00)	1.00	1.00
Additional timework other than this hospital				
yes	117 (60.00)	78 (40.00)	0.51 (0.33–0.78)^a^	1.88 (0.78–4.51)
no	70 (43.50)	91 (56.50)	1.00	1.00
Average working time				
≤ 8 h	71 (42.00)	98 (58.00)	2.25 (1.47–3.45)^a^	2.57 (1.17–8.301)^a^
> 8 h	116 (62.00)	71 (38.00)	1.00	1.00
Receive training				
Never	150 (50.00)	150 (50.00)	1.94 (1.07–3.54)^a^	2.31 (1.14–4.61)^a^
Have training	37 (66.10)	19 (33.90)	1.00	1.00
Read literatures				
Never	61 (44.50)	76 (55.50)	1.688 (1.09–2.59)^a^	1.47 (0.88–2.45)
Read sometimes and often	126 (57.60)	93 (42.50)	1.00	1.00

Key: ^a^ significant variables

## Discussion

The finding of this study shows that nurses who scored above the average score were 52.5%. The proportion of nurses who scored above the average value in the current study was lower than the studies conducted in Sweden (58.9%), Brazil (63.4%), and Addis Ababa Ethiopia 63.85% [22, 23, 26]. The difference might be due to the variation in the socio-economic and health care system of the countries.

In this study nurses correctly answered 57.9% of the eight-question items on the pressure ulcer classification and evaluation section. This finding is congruent with a study done in Iran which is 57% of all questions correctly answered by the respondents [27].

In the section of pressure ulcer prevention, a 33 item questionnaire was used, the current study result revealed that 62.4% question correctly answered by nurse respondents. This is in line with a study conducted in Iran and other countries with the result score 64.8% of pressure ulcer prevention questions answered correctly [17, 23, 28].

Knowledge of pressure ulcer prevention was also found to have a significant difference among gender groups. The proportion of subjects with poor knowledge was 44% lower among males than female nurses (AOR 0.56, 95% CI 0.36–0.88). This higher proportion of female nurses with a low level of knowledge might be related to the presence of additional responsibilities that females have in our society as it can limit the time they probably require to improve their professional knowledge. Furthermore, it could also limit their level of exposure which has been identified as a significant contributor for knowledge of nurses on pressure ulcer prevention. And this finding supported by previous studies conducted in different countries [29–31].

The present study revealed that nurses whose working time less than or equal to eight hours were 2.57 times much likely to have poor knowledge towards pressure ulcer prevention compared to nurses whose working time were more than eight hours (AOR 2.57, 95% CI 1.17–5.61). This could be since spending much time in the working environment will increase professional exposures for different activities in a hospital setup. As a result, their awareness level could be higher when compared to those who have a limited level of exposure for such types of medical cases. Besides, these repeated exposures may also give a chance to explore causes and possible prevention measures (10,15 30).

According to the findings of this study nurses who had no training on pressure ulcer prevention were 2.08 times high likely to have poor knowledge about pressure ulcer prevention compared to nurses who had training. (AOR 2.31, 95% CI 1.14–4.61) This result is in line with a study done in North West Ethiopia [19, 32].

In the current study statistically significant association was found among nurses who have a salary of fewer than 3201 birds were 3.4 times or (AOR 3.47, 95% CI 1.03–11.67), 3201–5250 birr was 3.9 times or (AOR 3.90, 95% CI 1.42–10.68) and those who got 5201–7800 birr were 2.9 times (AOR 2.94, 95% CI 1.04–8.30) high likely to have inadequate knowledge towards pressure ulcer compared to nurses with payment of more than 7800 birrs. This might be due to those nurses who got minimum salary may spend their free time working in another health facility to maximize their income this may lead to lack of time to read literature about pressure ulcer prevention and also it may be moral distress among nurses towards their salary could lead to not having good interest to know more about pressure ulcer prevention [14, 24, 33].

### Strengths and limitations

The strength of this study is using standard tools and this enhances the validity of the result and conclusion.

We use true/false questions in addition to Pieper-Zulkowski Pressure Ulcer knowledge test (PZ-PUKT) and we used mean score to define the level of knowledge. It might consider as a limitation because true/false questions are not in the form of a Likert scale and will affect the conclusion due to poor scoring of the mean. Likert scale is more recommended than true/false questions to calculate the mean.

## Conclusion

This study demonstrates that the Knowledge of nurses on pressure ulcer prevention is poor. The proportion of poor knowledge is higher among females, those who have low working hours and not having training were factors associated with a low level of knowledge, and low salary among nurses has a contribution of poor knowledge among nurses.

### Recommendation

Measures to improve nurse’s knowledge in pressure ulcer prevention needs to be conducted by giving priority for female nurses, those who have low working hours, and not having training on pressure ulcer prevention. Incorporating in the nursing curriculum, and formulating guidelines and improving the salary of nurses are some of the primary points to enhance nurses’ knowledge about pressure ulcer prevention. Furthermore, we recommend a multi-centered study to identify additional factors and effective interventions for addressing pressure ulcer prevention.

## Data Availability

The datasets used and/or analyzed during the current study available from the corresponding author on reasonable request.

## Abbreviations

AOR: Adjusted Odds Ratio
COR: Crude Odds Ratio
ENT: Ear Nose Throat
EPUAP: European Pressure Ulcer Advisory Panel
HAPU: Hospital-Acquired Pressure Ulcer
HUCSH: Hawassa University Comprehensive Specialized Hospital
ICU: Intensive Care Unit
NICU: Neonatal Intensive Care Unit
NPUAP: National Pressure Ulcer Advisory Panel
OPD: Out Patient Department
PU: Pressure Ulcer
PZ-PUKT: Pieper-Zulkowski Pressure Ulcer Knowledge Test
SNNPR: Southern Nations Nationalities and Peoples Region
SPSS: Statistical Package for Social Sciences
